# ForceGen: atomic covalent bond value derivation for Gromacs

**DOI:** 10.1007/s00894-017-3530-6

**Published:** 2017-12-06

**Authors:** Anthony Nash, Thomas Collier, Helen L. Birch, Nora H. de Leeuw

**Affiliations:** 1 Department of Physiology, Genetics, and Anatomy, University of Oxford, Oxford, UK; 2 Institute of Natural and Mathematical Sciences, Massey University, Palmerston North, New Zealand; 3 Institute of Orthopaedics and Musculoskeletal Science, Stanmore Campus, University College London, London, UK; 4 School of Chemistry, Cardiff University, Cardiff, UK

**Keywords:** Gromacs, Force values, Laplacian bond order, Molecular dynamics, Hessian

## Abstract

A large number of crystallographic protein structures include ligands, small molecules and post-translational modifications. Atomic bond force values for computational atomistic models of post-translational or non-standard amino acids, metal binding active sites, small molecules and drug molecules are not readily available in most simulation software packages. We present ForceGen, a Java tool that extracts the bond stretch and bond angle force values and equilibrium values from the Hessian of a Gaussian vibrational frequency analysis. The parameters are compatible with force fields derived using the second order tensor of the Hessian. The output is formatted with the Gromacs topology in mind. This study further demonstrates the use of ForceGen over the quantum mechanically derived structures of a small organic solvent, a naturally occurring protein crosslink derived from two amino acids following post-translational modification and the amino acid ligands of a zinc ion. We then derive Laplacian bond orders to understand how the resulting force values relate back to the quantum mechanical model. The parameterisation of the organic solvent, toluene, was verified using Molecular Mechanics simulations. The structural data from the simulation compared well with the quantum mechanical structure and the system density compared well with experimental values.

**Electronic supplementary material:**

The online version of this article (10.1007/s00894-017-3530-6) contains supplementary material, which is available to authorized users.

## Introduction

Molecular dynamics (MD) simulations are a powerful tool for investigating structures and biomolecular processes at the nano-scale [1]. However, atomistic force fields for protein structures complexed with small ligands,including drug-like molecules, or proteins that contain post-translational modifications are not always available. Force field terms for metal ions are a particular challenge, due to the variability in ligands and the variable oxidation state of the ions. Equally, the vast number of post-translationally modified amino acids is beyond the scope of an *off-the-shelf* MD packaged distribution.

Most force fields encode for a set of standard amino acids and perhaps half a dozen solvent molecules and ions. There is an absence of many small molecules, in addition to parameters for metal binding centres in enzymes, and parameters for post-translationally modified amino acids limiting their usefulness. Early methods of force field parameterisation were achieved using several numerically optimisation techniques, including the least-squares minimisation of fitting the energy of a molecular model to a quantum mechanical model [2], force values derived using a Genetic Algorithm [3, 4], and a modified simplex and Newton-Raphson algorithm [5–7]. However, these numerical techniques are time-consuming and computationally expensive.

Whilst the parameterisation of molecularly bonded terms is possible using AnteChamber [8] for the Amber MD suite and the Force Field Toolkit for CHARMM [9], there is a great deal of dependency on connectivity within organics. The parameterisation of metal complexes and features not represented in the standard library set may result in unassigned terms [10]. Furthermore, there is no widely available tool immediately compatible with the file format of a Gromacs compatible force field. Therefore, a solution to the derivation of bond parameters for small molecules would be invaluable for many Gromacs users.

Some research teams allude to the existence of software [4, 11]; however, the software is either no longer in the public domain or results are not readily transferable into Gromacs. Other tools, such as parafreq [10], although excellent in design, have not been suitable for high-throughput parameterisation of the organic libraries that our group has required.

Here, we present a software tool that calculates bond stretch and bond angle equilibrium constants (EqCs) and force values (FVs) based on Seminario’s method of fast diagonalisation of the QM Hessian matrix in Cartesian coordinates [11]. Although the derivation of dihedral force values are possible, they are not included in this release and the limitations of this software are made clear in the discussion. Force values are dependent on conformation, and it is the responsibility of the user to generate the optimised electronic structure fit for purpose and according to any experimental data at hand.

The software and sample files can be located at the address: https://sourceforge.net/projects/forcegen/. This approach resolves the dependency on how internal coordinates are defined and the sensitivity that internal coordinates have over intramolecular terms. This software requires the stored Hessian from a formatted checkpoint file of the Gaussian software package, and the output FV and EqC unit dimensions are immediately compatible with the file format of the Gromacs force field.

For this initial release the FV derivation from three different molecules for the AMBER energy expression, that is, an organic solvent, a metal binding centre and a glycation-mediated crosslink between two amino acids, are presented using a variety of QM methods. The Laplacian bond order (LBO) method is then applied to understand the underlying nature of the FVs. Finally, the bonded force values for the organic solvent, toluene, are used as force field parameters for the implementation in a molecular dynamics simulations within Gromacs. The bonded stretch and bonded angle distribution are compared against the QM electronic structure, and macromolecular properties are compared with experimental results.

### Approach

For the purpose of this study, we use the potential form of the ff99 Amber force field [12]. The bonded parameters make up part of the potential energy function:

Terms represent bond stretch, bond angle and dihedral bond angle, respectively. The coefficients, *k*_*b*_ and *k*_*𝜃*_ encode the force value for bond stretch and bond angle respectively. Equilibrium terms, *b*_*o*_ and *𝜃*_*o*_, can be defined using the optimised electronic structure geometry.

The Hessian matrix holds the second derivative of the potential energy with respect to change in atomic position [13]. This can be extracted from a Gaussian formatted checkpoint file in lower triangular matrix form. The 3*N* component reaction force *δ* **F**, due to small vibrational displacements *δ* **x** of the *N* atoms in a molecular system, can be expressed as:

$$[\delta\text{\textbf{F}}]=-[k][\delta\text{\textbf{x}}], $$

where [*k*], the Hessian, is a tensor of rank 2 matrix and has the dimension 3*N* ×3N defined by:

$$[k] = k_{ij} = \frac{\delta^2E}{\delta x_{i}\delta x_{j}}. $$

We utilised a method of pairwise atomistic interaction eigen-analysis to yield bond stretch and bond angle force value [11]. Diagonalisation of the Hessian, [*k*], returns eigenvalues corresponding to the three translational, three rotational and 3*N* − 6 vibrational normal modes. Each A-B atomic pair will have the 3×3 subset of [*k*]:

$$\left[\begin{array}{ll} \delta\text{\textbf{F}}_{Ax} \\ \delta\text{\textbf{F}}_{Ay} \\ \delta\text{\textbf{F}}_{Az} \end{array}\right] =\left[\begin{array}{lll} \frac{\delta^{2}E}{\delta x_{a}\delta x_{B}} & \frac{\delta^{2}E}{\delta x_{a}\delta y_{B}} & \frac{\delta^{2}E}{\delta x_{a}\delta z_{B}} \\ \frac{\delta^{2}E}{\delta y_{a}\delta x_{B}} & \frac{\delta^{2}E}{\delta y_{a}\delta y_{B}} & \frac{\delta^{2}E}{\delta y_{a}\delta z_{B}} \\ \frac{\delta^{2}E}{\delta z_{a}\delta x_{B}} & \frac{\delta^{2}E}{\delta z_{a}\delta y_{B}} & \frac{\delta^{2}E}{\delta z_{a}\delta z_{B}} \end{array}\right] \left[\begin{array}{ll} \delta x_{B} \\ \delta y_{B} \\ \delta z_{B} \end{array}\right]. $$

The diagonalisation of the matrix yields the eigenvalues $\lambda _{i}^{AB}$ and eigenvectors $v_{i}^{AB}$. The force value of the bond stretch can be calculated using:

$$k_{AB} = \sum\limits_{i = 1}^{3}\lambda_{i}^{AB}|u^{AB} \cdot v_{i}^{AB}|, $$

where *u* ^*AB*^ represents the normalised vector from atoms *A* to *B*. A similar approach is necessary for angular bonded force value of the triplet *∠* ABC. A subset from the complete Hessian of the atomic pairs A-B and B-C is identified:

$$\left[\begin{array}{ll} \delta\text{\textbf{F}}_{Ax} \\ \delta\text{\textbf{F}}_{Ay} \\ \delta\text{\textbf{F}}_{Az} \end{array}\right] =\left[\begin{array}{lll} \frac{\delta^{2}E}{\delta x_{A}\delta x_{B}} & \frac{\delta^{2}E}{\delta x_{A}\delta y_{B}} & \frac{\delta^{2}E}{\delta x_{A}\delta z_{B}} \\ \frac{\delta^{2}E}{\delta y_{A}\delta x_{B}} & \frac{\delta^{2}E}{\delta y_{A}\delta y_{B}} & \frac{\delta^{2}E}{\delta y_{A}\delta z_{B}} \\ \frac{\delta^{2}E}{\delta z_{A}\delta x_{B}} & \frac{\delta^{2}E}{\delta z_{A}\delta y_{B}} & \frac{\delta^{2}E}{\delta z_{A}\delta z_{B}} \end{array}\right] \left[\begin{array}{ll} \delta x_{B} \\ \delta y_{B} \\ \delta z_{B} \end{array}\right] , $$

$$\left[\begin{array}{ll} \delta\text{\textbf{F}}_{Cx} \\ \delta\text{\textbf{F}}_{Cy} \\ \delta\text{\textbf{F}}_{Cz} \end{array}\right] =\left[\begin{array}{lll} \frac{\delta^{2}E}{\delta x_{C}\delta x_{B}} & \frac{\delta^{2}E}{\delta x_{C}\delta y_{B}} & \frac{\delta^{2}E}{\delta x_{C}\delta z_{B}} \\ \frac{\delta^{2}E}{\delta y_{C}\delta x_{B}} & \frac{\delta^{2}E}{\delta y_{C}\delta y_{B}} & \frac{\delta^{2}E}{\delta y_{C}\delta z_{B}} \\ \frac{\delta^{2}E}{\delta z_{C}\delta x_{B}} & \frac{\delta^{2}E}{\delta z_{C}\delta y_{B}} & \frac{\delta^{2}E}{\delta z_{C}\delta z_{B}} \end{array}\right] \left[\begin{array}{ll} \delta x_{B} \\ \delta y_{B} \\ \delta z_{B} \end{array}\right]. $$

After diagonalisation of both matrices to yield eigenvectors and corresponding eigenvalues, bond angle force value can be calculated using:

$$\begin{array}{@{}rcl@{}} \frac{1}{k_{\theta}} &=& \frac{1}{d^{2}_{AB}{\sum}^{3}_{i = 1}\lambda^{AB}_{i}|u^{PA}\cdot v_{i}^{AB}|}\\ &&+ \frac{1}{d^{2}_{CB}{\sum}^{3}_{i = 1}\lambda^{CB}_{i}|u^{PC}\cdot v_{i}^{CB}|}, \end{array} $$

where *d*_*AB*_ and *d*_*CB*_ are distance vectors between atom B and the two displaced atoms, *A* and *C*:

$$u^{PA} = u_{N} u^{AB}; u^{PC} = u^{CB} u_{N}; u_{N} = \frac{u^{CB} \times u^{AB}}{|u^{CB} \times u^{AB}|}. $$

The bond stretch and bond angle EqCs are calculated using regular geometric expressions in 3D space.

## Methods

### Quantum chemistry methods

Electronic structure calculations and vibrational frequency calculations were performed using Gaussian 09 C.01 [14]. The convergence criterion for maximum force, root mean square (RMS) force, maximum displacement and RMS displacement remained the default. Vibrational frequency analysis was performed for each optimised structure to verify that an energy minimum had been found and for output of the Hessian.

Each structure was optimised using three different QM methods, Hartree-Fock (HF), the post-Hartree Fock *ab initio* Møller-Plesset perturbation theory to second order (MP2) and the hybrid density functional theory (DFT) based, B3LYP, WB97XD and B3PW91. A 6-31+G(p) basis set was applied to the toluene and MOLD systems, whilst the effective core potential based basis set LANL2DZ was applied to the zinc binding centre system.

Finally, intuitive bond orders between nuclear centres were calculated from the electronic landscape using the LBO [15] implementation within the Multiwfn software suite version 3.3.7 [16].

### Force value derivation

ForceGen can be downloaded from SourceForge as an executable Jar and requires Java 1.8 or later. It can be launched from a secure shell using X11 forwarding within a command/terminal window or on the desktop. Instructions for use are readily available in the *Instructions* menu (SM Fig. 1). The source code has been made available under the GNU General Public License. The Jar library packages Jama-1.0.3 Java Matrix Package and Commons-maths3-3.5 Apache Commons Maths are only required in order to make changes to the Java source code; links can be found in the README file.

The derivation of bonded terms require the following steps; firstly, the molecule of interest is subjected to an electronic structure optimisation, followed by vibrational frequency analysis using the Gaussian software. Ideally, the method and basis set used ought to reproduce available experimental inter-atomic distances and angles to an acceptable degree. The vibrational frequencies are as accurate as the choice of method and basis set, and therefore FVs and EqCs can be tuned at the quantum mechanical level before implementing bonded parameters into a mechanical model (MM). Harmonic vibrational frequencies are typically larger than those observed experimentally [17]. This overestimation can be scaled during a FV calculation by supplying an appropriate scalar within the ForceGen GUI.

**Fig. 1:**
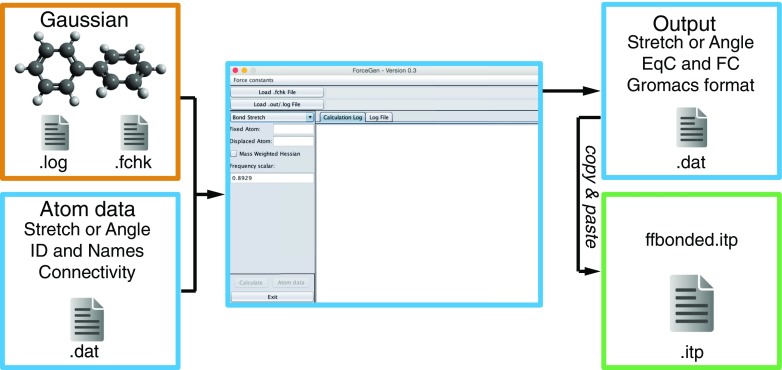
Schematic of work flow. Orange represents stages requiring Gaussian; blue, the use of ForceGen; and green, the implementation into GROMACS

Successful execution of Gaussian yields a binary checkpoint file and logged output file, both of which are loaded into the ForceGen GUI once the checkpoint file has been converted into a formatted checkpoint file, that is human readable (Fig. 1, left). The software can be executed in one of two ways; if the user requires only a small number of bond stretch and/or bond angle FVs and EqCs, corresponding atom IDs can be entered by hand. Otherwise, a large set of bonded and angular corresponding atom IDs, alongside unique force field atom names, can be read in via a user defined text file (Fig. 1, middle). Each atom ID pair or triplet will be used to derive a bond stretch or bond angle, respectively, the result of which is then saved to a text file (Fig. 1, middle) which can then be copied to the bonded parameter file of the Gromacs force field (Fig. 1, right).

To illustrate these steps, we present three cases. The first, toluene, represents small organic solvents. Given its size, all bond stretch and bond angle FVs and EqCs are presented within the main text. Toluene is a common organic solvent, for which quantum mechanical bonded properties have already been established and experimental macroscropic properties have been well documented [18]. The second case, the methylglyoxal lysine dimer (MOLD) [19], demonstrates use for amino acids that have been post-transnationally modified and cross-linked together. ForceGen is capable of providing a complete set of bond stretch and bond angle FVs and EqCs for various post-translational modifications, such as glycation, glycosylation, phosphorylation and lipidation. Although ForceGen is not restricted by the number of atoms, the user may be restricted by the computational demand during QM optimisation and frequency calculations of large structures. The third and final case is a zinc ion coordinated by the functional groups of four ligand side chains from three histidine and one aspartic acid. Zinc ion binding centres are common within the human proteome, for example the large family of matrix metalloproteinases, in which our group has particular interest [20]. Only the FVs and EqCs necessary to establish a bonded metal model are reported.

For clarity, two of the three sets of bond parameters (FVs, EqCs and LBOs) for toluene have been placed in the supplementary material, along with all bond parameters (FVs, EqCs and LBOs) for MOLD and the zinc binding centre. However, they will be referred to throughout the main text.

### MD Simulations

The MD simulation set-up of the neat liquid toluene was parameterised according to the method described by Fioroni and Vogt [18], and apart from notable differences, the methods will not be reproduced here. The recent version of Gromacs, 5.1.4, was utilised. A total of 216 toluene molecules were uniformly distributed within a cubic box. The force values and equilibrium values of bond stretch and bond angle terms were obtained from B3LYP/6-31+G(d) electronic structure calculations, as described above and presented below. The atomic partial charge values were obtained by submitting the output file from the electronic structure calculation to the R.E.D server [21]. Both the benzene group of phenylalanine and the terminal methyl group of isoleucine from the Amber99sb force field were used for the initial sigma and epsilon values of the Lennard Jones potential. Both sets of parameters were then adjusted until the density a neat liquid toluene was in close agreement with the value of the experimental density.

## Results

### Force value derivation

The electronic structure optimisation and vibrational frequency analysis for toluene was performed using the three methods of theory; HF, B3LYP (Fig. 3, a), and MP2, along with the 6-31+G(d) basis set. A complete set of bond stretch and bond angle FVs and ECs from the B3LYP QM model is presented in Table 1. The parameters based on MP2 and HF calculations are presented in the supplementary material (SM Table 1), although, they will be discussed in the main text.

**Table 1:** Bond stretch and bond angle force values and equilibrium values for a toluene molecule calculated using the hybrid DFT B3LYP

Bond angle	*𝜃*_*o*_ (^∘^)	*k*_*𝜃*_ (kJ/mol/rad^2^)	Bond stretch	*b*_*o*_ (nm)	*k*_*b*_(kJ/mol/nm^2^)
C_1_-C_2_-C_3_	120.777	178.619	C_1_-C_2_	0.151	100154.048
C_1_-C_2_-C_4_	121.054	178.556	C_2_-C_3_	0.140	133511.266
C_2_-C_3_-C_5_	121.046	248.267	C_2_-C_4_	0.140	134946.337
C_2_-C_4_-C_6_	121.059	248.476	C_3_-C_5_	0.140	165277.428
C_3_-C_2_-C_4_	118.160	248.912	C_4_-C_6_	0.140	163709.594
C_3_-C_5_-C_7_	120.176	241.405	C_5_-C_7_	0.140	144447.738
C_4_-C_6_-C_7_	120.162	241.225	C_6_-C_7_	0.140	145818.268
C_5_-C_7_-C_6_	119.397	234.433	C_1_-H_1_	0.110	101760.537
H_1_-C_1_-H_2_	107.379	237.011	C_1_-H_2_	0.110	76862.207
H_1_-C_1_-H_3_	107.929	148.157	C_1_-H_3_	0.110	129283.846
H_2_-C_1_-H_3_	107.182	148.507	C_3_-H_4_	0.109	156322.742
H_1_-C_1_-C_2_	111.470	111.744	C_4_-H_5_	0.109	156391.043
H_2_-C_1_-C_2_	111.166	139.824	C_5_-H_6_	0.109	158382.690
H_3_-C_1_-C_2_	111.502	189.814	C_6_-H_7_	0.109	158391.619
C_2_-C_3_-H_4_	119.377	79.573	C_7_-H_8_	0.109	158994.145
C_2_-C_4_-H_5_	119.371	79.215			
C_3_-C_5_-H_6_	119.753	79.310			
C_4_-C_6_-H_7_	119.733	79.252			
C_5_-C_3_-H_4_	119.578	79.370			
C_6_-C_4_-H_5_	119.570	79.140			
C_5_-C_7_-H_8_	120.287	80.156			
C_6_-C_7_-H_8_	120.316	80.125			
C_7_-C_6_-H_7_	120.105	78.299			
C_7_-C_5_-H_6_	120.071	78.445			

**Fig. 2:**
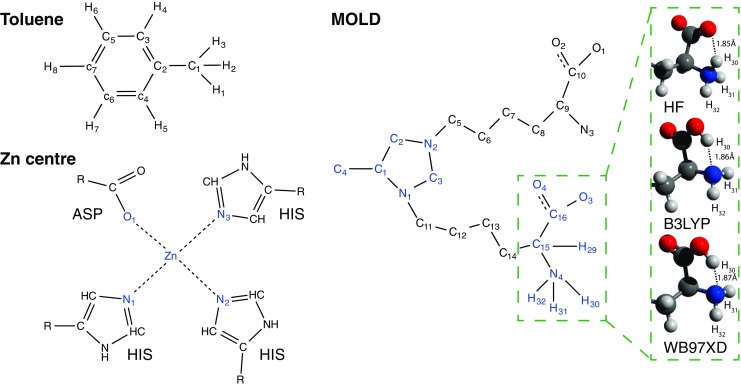
The structural representation of the three use-cases, toluene, MOLD and a zinc ligand model. Those atoms indicated in blue have an entry in a corresponding table in the main text. Inset: the backbone of MOLD as visualised from the optimised electronic structures

**Fig. 3:**
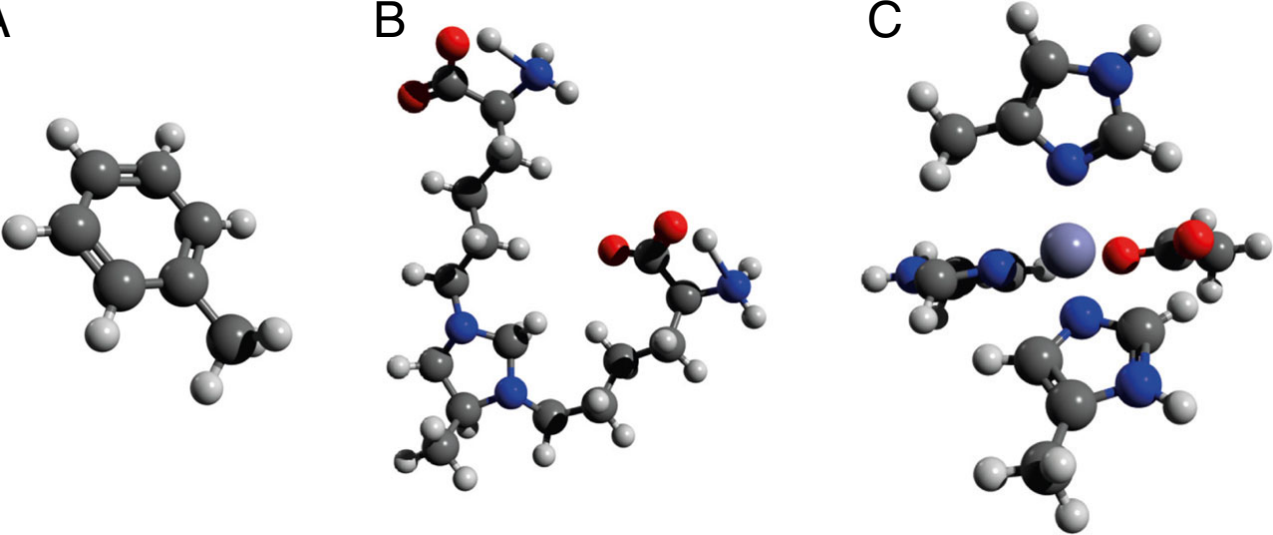
Final optimised structure using the B3LYP method of **a** toluene, **b** MOLD, and **c** a zinc-binding centre

Given the size of MOLD the MP2 method of theory was substituted by the hybrid WB97XD functional. For the sake of clarity, we have only reported bonded features from the cross-link ring structure and one of the two backbones (SM Table 2). The results are sufficient to illustrate the application of ForceGen on modified protein structures. A complete list of FVs and EqCs is available for download and the final optimised structure using the B3LYP method can be seen in Fig. 3, b. Our initial model employed two sets of charged backbone termini: a positively charged amine and a negatively charged carboxylic acid. The final structure using both hybrid DFT approaches resulted in proton transfer from the backbone nitrogen to the electronegative backbone carboxylic acid (see the inset in Fig. 2). This was not observed in the HF model. The discrepancy in proton nuclei association can be seen in FV of the pair N_4_-H_30_, illustrating the effect on the FV when paired atoms disassociate or from the erroneous input from a user. This is very apparent when the atom IDs of an unrealistic bond stretch or bond angle are supplied.

**Table 2:** Laplacian bond order for toluene using the B3LYP method

Bond	LBO
C_1_-C_2_	1.072374
C_2_-C_3_	1.510786
C_2_-C_4_	1.516674
C_3_-C_5_	1.549244
C_4_-C_6_	1.544522
C_5_-C_7_	1.548748
C_6_-C_7_	1.554352
C_1_-H_1_	0.781929
C_1_-H_2_	0.770005
C_1_-H_3_	0.782038
C_3_-H_4_	0.796466
C_4_-H_5_	0.797323
C_5_-H_6_	0.803375
C_6_-H_7_	0.802899
C_7_-H_8_	0.799741

The bond orders are intuitive: 1 indicates a single bond, 2 a double bond and 1.5 approximates delocalised open shell covalence

The zinc ligand model was optimised using the LANLDZ2 effective core potential basis-set with a HF, B3LYP ((Fig. 3, c)) and B3PW91 level of theory. The inner-shell electrons are represented using an electrostatic potential, whilst the valence electrons are treated explicitly using a double-zeta Pople-type basis set [22]. The optimised structure presented a Zn^2+^ ion coordinated with four ionic ligand pairs; N_1_-Zn, N_2_-Zn, N_3_-Zn, and O_1_-Zn. The interatomic distances between ligand and metal centre and the bond angles between ligand-metal-ligand (SM Table 3), were all in close agreement and the magnitude of the corresponding FVs was significantly smaller than those presented in non-metal to non-metal covalent bonds.

### Laplacian bond order

We use an approach that utilises the Laplacian over a *fuzzy*-defined region between nuclear centres of the electronic environment to yield intuitive bond orders in-line with presented bond stretch FVs.

The Laplacian Bond Order (LBO), as implemented within the Multiwfn software suite by Lu and Chen [15], presents an intuitive representation of bond order between nuclear centres from regions that do not correspond to a closed-shell region. It has been shown that the LBO relates to the bond vibrational frequency and bond dissociation energy and we can therefore identify a correlation between the FVs in any one molecule with their corresponding LBO. This is particularly useful for groups of identically-bonded atom types exhibiting varying bond stretch FVs, such as hydrogen-carbon pairs in methyl groups.

The LBO is defined as:

$$\text{LBO}_{A,B} = 10\times{\int}_{\nabla^{2}\rho<0}w_A(\text{\textbf{r}})w_B(\text{\textbf{r}})\nabla^{2}\rho(\text{\textbf{r}})\text{d\textbf{r}}, $$

where the integration is over the negative electron Laplacian, ∇^2^ *ρ*(**r**), using a *fuzzy* overlap space between two nuclear centres defined by the product *w*_*A*_(**r**)*w*_*B*_(**r**). Positive ∇^2^ *ρ* values represent areas of closed and depleted electron space, notably non-covalent interactions.

The B3LYP optimised structure of toluene describes a bond order approximating that of an ideal single bond for the C_1_-C_2_ pair (Table 2), whilst HF and MP2 methods over-estimate the bond order (SM Table 4). The *π*-bonds, resulting in electron delocalization about the carbon atoms of the benzene ring, are suitably reported as a bond and a half from the B3LYP optimisation, yet both the HF and MP2 method result in a bond order a little under two bonds. Between carbon-hydrogen atom pairs over all three methods, the difference in electronegativity establishes a slight polarity in the covalent bond, resulting in a drop in the LBO value.

**Fig. 4:**
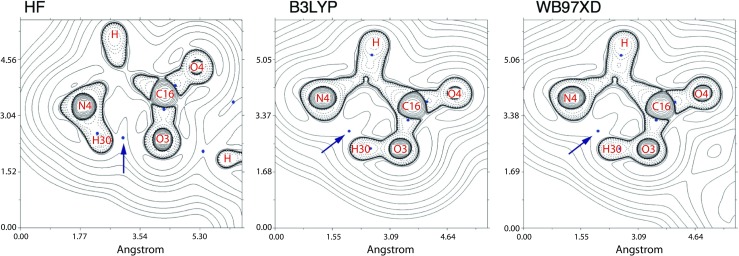
Contour plots of the Laplacian of the electron density (∇^2^ *ρ*) defined on a plane between the nuclear centres of atoms O_3_, N_4_ and H_30_ from the MOLD backbone calculated over three levels of theory. Regions of locally depleted electron density (∇^2^ *ρ* > 0) can be seen between positive (solid) contours, and areas of locally concentrated electron density (∇^2^ *ρ* < 0) between dashed contours; this is the space about and between nuclear centres that have formed covalent bonds. Bond critical points in locally depleted regions of electron density can be used to identify non-covalent bonds (identified by an arrow)

In the case of the MOLD crosslink, all three QM methods consistently report bond orders for carbon-nitrogen pairs a little under that of a single bond, suggesting the pull on the valence electrons of the carbon from a difference in electro negativity (SM Table 5). With the exception of the over-estimation attained from the HF method, the LBO value of carbon to carbon closely resembles a single bond. The LBO of the carbonyl group on the crosslink backbone indicates that a significant portion of the electronic charge is centred over the C_16_ - O_4_ bond. Both WB97XD and B3LYP report a drop in the LBO value of C_16_ - O_3_ as a result of the proton transfer from the amide. Where the proton had shifted from the amine group the neighbouring electron landscape was analysed for bond critical points (BCP) in regions of depleted electron density. The HF method produces a non-covalent BCP between the hydrogen (H_30_) on the positively charged amine with the oxygen (O_3_) on the negatively charged carboxylic acid (Fig. 4, a). Both hybrid DFT methods describe the shift in proton as mentioned earlier in additional to non-covalent BCP between N_4_ and H_30_ (Fig. 4, b and c).

Finally, all three QM methods yield similar LBO values between each ligand and zinc ion pair (SM Table 6). The dearth in each bond order is a clear indication of the ionic nature of a non-metal to metal bond.

### Molecular dynamics implementation of toluene

The force values and equilibrium values for the bond stretch and bond angle of toluene were calculated using ForceGen from a B3LYP/6-31+G(d) electronic structure calculation. Those values presented in Table 1 were added to the ffbonded.itp file within the Gromacs topology subdirectory and a complete set of new atom types were added to atomtype.dat. A set of dihedral angles were provided using those from similar structures in the Amber99SB force field [12]. The initial sigma and epsilon values of the Lennard-Jones function were acquired from the benzene of phenylalanine and the terminal methyl group from isoleucine. A neat liquid of toluene was constructed using 216 toluene molecules in a cubic unit cell under periodic boundary conditions. The bond lengths and bond angles were equilibrated using the steepest descent method until the maximum force in the system reached below 400 kJ mol^− 1^ nm^− 1^. A 20 ps simulation using the NVT ensemble was performed, by applying the Berendsen thermostat [23], to stabilise a temperature of 298 K. The velocities were preserved and the system evolved for 1 ns using the NPT ensemble by applying the Berendsen pressure-coupling scheme [23] and the Nośe-Hoover thermostat [24] before extending by 5 ns for a production simulation using the Parrinello–Rahman pressure-coupling [25].

**Fig. 5:**
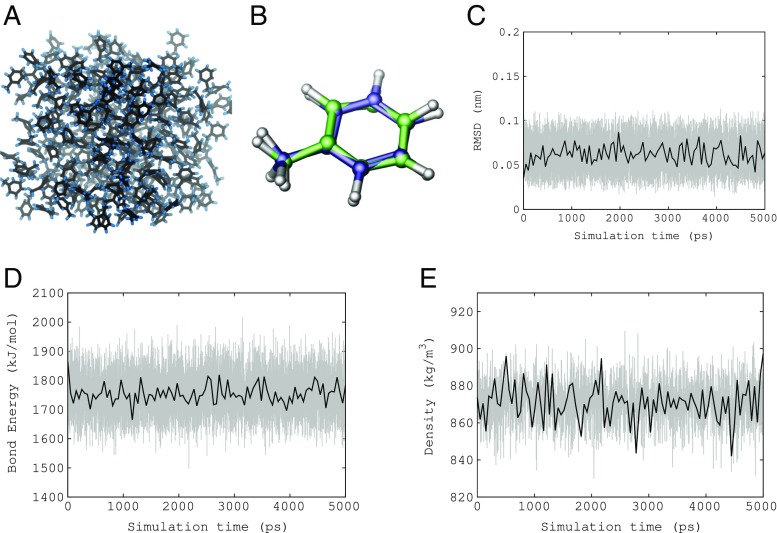
Results from the 5 ns MD simulation: **a** a visual representation of the final frame, (2) the superposition of the average structure taken from a random toluene molecule (green) over the QM optimised structure (blue), **c** the RMSD of the single bonded structure, **d** the total bond energy and **e** the system density

From observation, the trajectory, of which a snapshot can be seen in Fig. 5, a, demonstrated an amorphous distribution of toluene molecules. The pressure, temperature, potential energy and RMSD of the complete system converged shortly after the start of the 5 ns production simulation. To measure the stability of the structure compared with the QM minimum structure, a single toluene molecule was randomly picked. The superposition as seen in Fig. 5, b, reveals a very close fit to the QM structure. The structural RMSD (Fig. 5, c) and the bond energy (Fig. 5, d) of the same molecule was measured over the duration of the trajectory and clearly demonstrates a very stable bonded configuration.

The distribution of bond length and bond angle measurements from 216 toluene molecules was calculated over the 5 ns simulation (Table 3) and compared against those of the QM optimised structure. The force values for bond stretch reproduce the desired bond lengths to such a degree that recording the error would be insignificant. The bond angles were able to reproduce those of the QM structure within a degree of statistical error. Finally, the macroscopic density was reported to be 869 ± 9 kg/m^3^ (Fig. 5, e), which is in good agreement with the experimental value of 862 kg/m^3^ at the lower temperature of 293.15 K [27].

**Table 3:** The average bond length and bond angle of toluene over the 5 nm simulation alongside QM structure calculations using the B3LYP method

Bond angle	QM (^∘^)	MD* (^∘^)	Bond length	QM (nm)	MD (nm)
C_1_-C_2_-C_3_	120.777	120.499 ± 5.317	C_1_-C_2_	0.151	0.151
C_1_-C_2_-C_4_	121.054	120.139 ± 5.467	C_2_-C_3_	0.140	0.140
C_2_-C_3_-C_5_	121.046	118.151 ± 4.418	C_2_-C_4_	0.140	0.140
C_2_-C_4_-C_6_	121.059	118.135 ± 4.430	C_3_-C_5_	0.140	0.140
C_3_-C_2_-C_4_	118.160	114.956 ± 4.210	C_4_-C_6_	0.140	0.140
C_3_-C_5_-C_7_	120.176	117.971 ± 4.276	C_5_-C_7_	0.140	0.140
C_4_-C_6_-C_7_	120.162	117.971 ± 4.329	C_6_-C_7_	0.140	0.140
C_5_-C_7_-C_6_	119.397	116.543 ± 4.276	C_1_-H_1_	0.110	0.110
H_1_-C_1_-H_2_	107.379	107.176 ± 5.633	C_1_-H_2_	0.110	0.110
H_1_-C_1_-H_3_	107.929	107.406 ± 6.789	C_1_-H_3_	0.110	0.110
H_2_-C_1_-H_3_	107.182	106.706 ± 6.760	C_3_-H_4_	0.109	0.109
H_1_-C_1_-C_2_	111.470	111.419 ± 7.275	C_4_-H_5_	0.109	0.109
H_2_-C_1_-C_2_	111.166	111.214 ± 6.784	C_5_-H_6_	0.109	0.109
H_3_-C_1_-C_2_	111.502	111.477 ± 6.039	C_6_-H_7_	0.109	0.109
C_2_-C_3_-H_4_	119.377	120.764 ± 7.447	C_7_-H_8_	0.109	0.109
C_2_-C_4_-H_5_	119.731	120.772 ± 7.371			
C_3_-C_5_-H_6_	119.753	119.089 ± 7.547			
C_4_-C_6_-H_7_	119.733	118.286 ± 7.824			
C_5_-C_3_-H_4_	119.578	117.450 ± 7.686			
C_6_-C_4_-H_5_	119.570	117.416 ± 7.616			
C_5_-C_7_-H_8_	120.287	120.497 ± 7.541			
C_6_-C_7_-H_8_	120.316	119.492 ± 7.710			
C_7_-C_6_-H_7_	120.105	120.107 ± 7.541			
C_7_-C_5_-H_6_	120.071	119.287 ± 7.785			

The standard deviation of the bond length was less than 0.0001

## Discussion

Using ForceGen the FVs and EqCs for toluene, MOLD and a zinc-ligand model were generated. Each structure was optimised using three different QM methods to illustrate how variance between force value output is a result of the QM methods and basis set applied, and not erroneous artefacts introduced during development. Confidence with the FVs can be established by comparing the derived values to electronic environment properties such as bond order, in particular, the Laplacian bond order. Although, not every FV can be clearly understood using covalent-electron based techniques we implemented the derived parameters of toluene into an MM model to compare against QM structural data and macroscopic experimental properties.

The equilibrium value (bond length and bond angle) were validated by measuring the distance between each bond pair and bond angle between each bond triplet from the optimised structure and comparing it to the output generated by ForceGen.

The set of derived covalent bond stretch FVs, across all three methods per use case compare well with the magnitude of bond stretch FVs already present in the latest Amber force field for a Gromacs implementation. Equally, bond angle FVs presented here show a similar trend in FV magnitude to those already present. The bond stretch FVs for toluene (Table 1) report a discrepancy between values associated with all three hydrogen to carbon bonds of the mono-methyl group. There is also a significant drop from the FVs of the carbon to carbon bonds in the benzene ring, compared with the benzene carbon to methyl carbon bond. Although in the latter this is a result of the close association between benzene carbon atoms due to the delocalised *π*-bonds, in the former, although the FVs are not consistent, their difference corresponds to the difference in bond order (Table 2, SM Table 4). The C_1_-H_2_ FV from the HF calculation is significantly lower than either neighbouring carbon to hydrogen atomic pair. This is also seen with the B3LYP method, which similarly presents the case for a greater difference between the two neighbouring C-H pairs. Whilst a calculation using MP2 yields a smaller FV on the C_1_-H_3_ bond, the remaining two bonds in the methyl group are similar. Equivalent bonded atoms in the benzene, C_3_-H_4_ and C_4_-H5, and C_5_-H_6_ and C_6_-H_7_, return almost identical bond stretch FVs consistent with very similar bond orders.

There is considerable variation in bond stretch FVs across all three methods within the ring structure of MOLD (SM Table 2). This comes as little surprise given that each bond pair has a unique chemical environment with the exception of nitrogen atoms N_1_ and N_2_ which pair with a carbon from the lysine-derived aliphatic side chains. Both hybrid DFT methods yield similar bond stretch FVs between the C_15_-C_16_ on the backbone, and the corresponding bond order is very close to a single bond (SM Table 5). The HF method over-estimates the single bond whilst returning a significant drop in the associated FV. This is similar to the relationship between C_1_-C_2_ from all three methods as seen before.

A comparison in bond connectivity between QM methods on the carboxylic acid backbone is not as straightforward. Both hybrid DFT methods return an increase in C_16_-O_3_ bond length as the optimisation of the structures results in a proton detachment from the charged amide group, negating the delocalisation of electrons. Given the detachment of H_30_ the affinity for protons on N_4_ can only be compared between the two hybrid DFT methods. The N_4_-H_30_ FV is significantly greater than either neighbouring nitrogen-hydrogen pair. Interestingly, the corresponding bond order is noticeably less than those belonging to either N_4_-H_31_ or N_4_-H_32_. Despite a drop in bond order, the increase in force value could be attributed to a contribution from neighbouring non-bonded interactions as per the presence of a bond critical point between H_30_ and O_3_. The difference in bond stretch FV between N_4_-H_31_ and N_4_-H_32_ from both hybrid DFT methods corresponds well with the difference in bond order.

The derivation of force values from three different optimised structures of MOLD has yielded values indicative of the LBO and bonding neighbours. The bond stretch FV of the carbon to carbon pairs, C_1_-C_2_ and C_1_-C_4_, are in agreement with their LBO values. Both hybrid DFT methods approximate a single bond with FVs very similar between pairs. However, the HF method returns a significant difference in FV between the two bonds, yet the bond order still reflects this.

The smaller bond stretch FVs in the zinc ligand model (SM Table 3), coupled with small bond orders (SM Table 6), is indicative of highly ionic interactions. With such small values the difference in FV from each QM method is insignificant. It is interesting to note how the slight difference in electronegativity presents a smaller LBO value from O_1_-Zn than between either three nitrogen ligands and the zinc ion.

The implementation of FVs and EqCs of toluene into an MM model resulted in a stable atomistic representation. The Lennard-Jones parameters were tuned using short NPT simulations until the density from a final production simulation was within close agreement of experimental values [18]. The RMSD of a single toluene molecule displayed little in structural deviation, which compares well with the strict distribution of MM bond lengths and bond angles. The superposition of the MM model over a QM model and a comparison between the length and angle distributions of both levels suggests that the MM model varies little from the original QM model. Finally, bonded potential energies during the 5 ns production run deviated little, again indicative of a stable bonded arrangement. The force values and equilibrium values derived from ForceGen show promise in light of the stable structure and similar system densities presented here.

There are a number of noted limitations to the software. While the extrapolation of dihedral angles from the diagonalisation of the Hessian is possible, the harmonic approximation is poor and they are better determined by scanning over the potential energy surface [10]. Secondly, comparing the normal mode frequencies of a QM model to those of a MM model is a common technique but as Seminario’s approach does not decouple bonded interactions within the Hessian from non-bonded contributions, reproducing frequencies becomes challenging and at worst misleading.

Finally, normal mode analysis of force values using the documented approach, have been shown to frequently fail to reproduce the normal modes from the QM spectrum[10], owing to the poor reproducibility of low-frequency vibrational modes [26]. Therefore, we described the variation in FVs using an intuitive bond order and validated the FVs through comparing their implementation in an MD simulation against QM and experimental macroscopic values.

## Summary

In this study, we have presented a software tool used to derive bond stretch and bond angle force values, in addition to distance and angular equilibrium values, for a force field compatible with the second order tensor of a QM Hessian. The software is a stand alone feature and provides the user with bonded data necessary for the construction of small molecules in a format compatible with the Gromacs topology files.

At the time of writing, we have made public our first release of ForceGen. In addition, our ongoing studies on modelling combustible materials have used this method and have accurately reproduced experimental bulk properties. In future releases we aim to implement a graphical representation of the molecule, providing the user with a means of selecting individual atoms using a mouse or with a touch screen device rather than entering atom IDs manually.

## Electronic supplementary material

Below is the link to the electronic supplementary material.

(PDF 709 KB)
