# Supplementary material for: ForceGen: atomic covalent bond value derivation for Gromacs
Source: J Mol Model. 2017 Dec 6;24(1):5. doi: 10.1007/s00894-017-3530-6 (PMC5719132; doi:10.1007/s00894-017-3530-6)
Supplement: Supplementary file 1 — (PDF 709 KB) [file 894_2017_3530_MOESM1_ESM.pdf]

Supplementary Information: ForceGen: atomic covalent bond constant derivation for Gromacs.

Anthony Nash,[†] Thomas Collier,[‡] Helen L Birch,[¶] and Nora H de Leeuw[*,†,§]

†*University College London, Department of Chemistry, London, WC1E 6BT, UK*

‡*Massey University, New Zealand*

¶*University College London, Institute of Orthopaedics and Musculoskeletal Science, Royal National Orthopaedic Hospital, Stanmore, HA7 4LP, UK*

§*Cardiff University, School of Chemistry, Cardiff, CF10 3AT, UK*

E-mail: a.nash@ucl.ac.uk

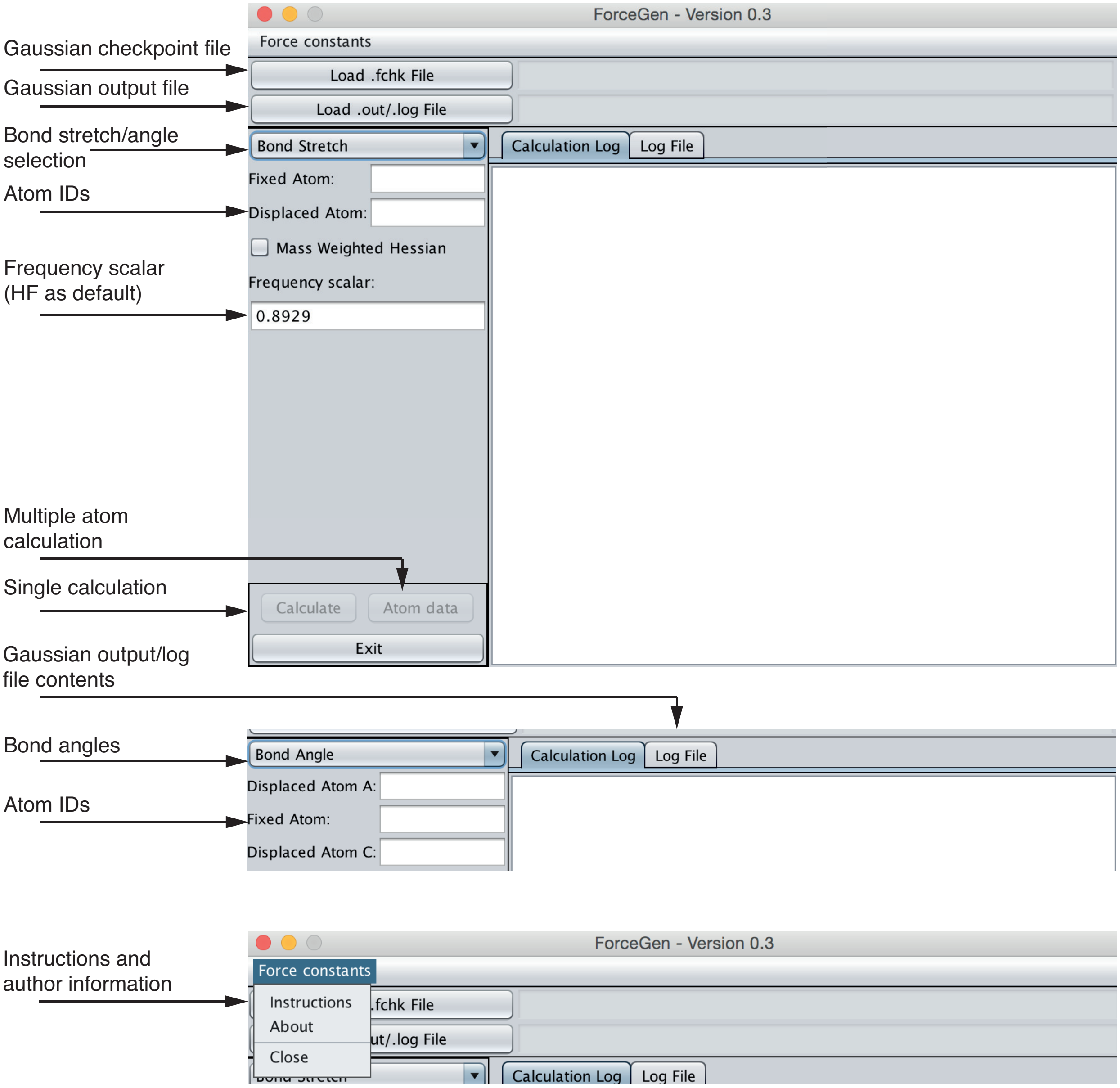

Figure 1: Screenshots of the bond stretch, bond angle and menu screen.

Bond stretch atom IDs with atom types

```
Stretch
47 5 N GC13
5 6 GC13 GO1
5 4 GC13 GC1
4 12 GC1 GH1
4 3 GC1 GN5
3 21 GN5 GH2
3 1 GN5 C
4 7 GC1 GC2
7 8 GC2 GC3
8 9 GC3 GC4
9 10 GC4 GC5
10 11 GC5 GN1
7 13 GC2 GH3
7 14 GC2 GH4
8 15 GC3 GH5
8 16 GC3 GH6
9 17 GC4 GH7
9 18 GC4 GH8
10 19 GC5 GH9
10 20 GC5 GH10
```

Bond stretch (with eq length) force constants - gromacs compatible

GROMACS – atom types internal to Gromacs (supplied by user)

i	j	func	len_eq(nm)	FC (kJ/mol/nm^2)
N	GC13	1	0.134	194129.527
GC13	GO1	1	0.121	247488.448
GC13	GC1	1	0.153	61750.248
GC1	GH1	1	0.108	159269.846
GC1	GN5	1	0.145	116409.974
GN5	GH2	1	0.100	201207.446
GN5	C	1	0.136	167023.302
GC1	GC2	1	0.154	75726.923
GC2	GC3	1	0.154	77503.248
GC3	GC4	1	0.153	161472.264
GC4	GC5	1	0.154	75284.156
GC5	GN1	1	0.145	113535.046
GC2	GH3	1	0.109	86270.049
GC2	GH4	1	0.109	81149.230
GC3	GH5	1	0.109	77043.771
GC3	GH6	1	0.108	31021.950
GC4	GH7	1	0.109	133280.148
GC4	GH8	1	0.109	43397.133
GC5	GH9	1	0.109	38652.885
GC5	GH10	1	0.108	122297.001

Bond angle atom IDs with atom types

```
ANGLE
52 47 5 CA N GC13
53 47 5 H N GC13
47 5 6 N GC13 GO1
47 5 4 N GC13 GC1
6 5 4 GO1 GC13 GC1
5 4 12 GC13 GC1 GH1
5 4 7 GC13 GC1 GC2
5 4 3 GC13 GC1 GN5
4 7 8 GC1 GC2 GC3
4 3 21 GC1 GN5 GH2
4 3 1 GC1 GN5 C
12 4 7 GH1 GC1 GC2
12 4 3 GH1 GC1 GN5
3 1 2 GN5 C O
3 1 57 GN5 C CA
21 3 1 GH2 GN5 C
4 7 13 GC1 GC2 GH3
4 7 14 GC1 GC2 GH4
3 4 7 GN5 GC1 GC2
13 7 14 GH3 GC2 GH4
13 7 8 GH3 GC2 GC3
14 7 8 GH4 GC2 GC3
8 9 10 GC3 GC4 GC5
7 8 15 GC2 GC3 GH5
7 8 16 GC2 GC3 GH6
15 8 16 GH5 GC3 GH6
15 8 9 GH5 GC3 GC4
16 8 9 GH6 GC3 GC4
8 9 10 GC3 GC4 GC5
```

Bond angle (with eq angle) force constants - gromacs compatible

GROMACS – atom types internal to Gromacs (supplied by user)

i	j	k	func	deg_eq(degree)	FC (kJ/mol/rad^2)
CA	N	GC13	1	121.758	309.827
H	N	GC13	1	117.909	146.613
N	GC13	GO1	1	122.832	283.822
N	GC13	GC1	1	117.087	424.555
GO1	GC13	GC1	1	119.915	338.460
GC13	GC1	GH1	1	105.620	208.972
GC13	GC1	GC2	1	109.621	509.974
GC13	GC1	GN5	1	111.971	256.997
GC1	GC2	GC3	1	113.614	195.721
GC1	GN5	GH2	1	117.216	103.950
GC1	GN5	C	1	120.147	269.623
GH1	GC1	GC2	1	110.078	244.702
GH1	GC1	GN5	1	109.690	167.903
GN5	C	O	1	121.597	229.835
GN5	C	CA	1	115.731	189.496
GH2	GN5	C	1	117.581	121.311
GC1	GC2	GH3	1	108.616	264.451
GC1	GC2	GH4	1	108.507	313.210
GN5	GC1	GC2	1	109.786	271.144
GH3	GC2	GH4	1	106.380	126.597
GH3	GC2	GC3	1	110.466	173.209
GH4	GC2	GC3	1	108.989	243.480
GC3	GC4	GC5	1	114.479	261.649
GC2	GC3	GH5	1	109.188	104.360
GC2	GC3	GH6	1	109.878	330.892
GH5	GC3	GH6	1	106.348	135.044
GH5	GC3	GC4	1	110.316	207.938
GH6	GC3	GC4	1	109.027	217.524
GC3	GC4	GC5	1	114.479	261.649

Figure 2: Example content of bond stretch input file, bond angle input file, and their corresponding output, ready for immediate implementation into Gromacs.

Table 1: Bond stretch and bond angle force constants and equilibrium constants for a toluene molecule.

Bond stretch	HF		MP2	
	b_o (nm)	k_b (kJ/mol/nm^2)	b_o (nm)	k_b (kJ/mol/nm^2)
C_1-C_2	0.151	117353.442	0.151	109366.164
C_2-C_3	0.139	150796.992	0.140	150015.724
C_2-C_4	0.139	150676.708	0.140	150017.034
C_3-C_5	0.139	181861.382	0.140	175061.010
C_4-C_6	0.139	182085.289	0.140	175059.713
C_5-C_7	0.139	227825.122	0.140	160179.358
C_6-C_7	0.139	227725.070	0.140	160180.282
C_1-H_1	0.108	99842.951	0.109	128829.737
C_1-H_2	0.109	48354.522	0.109	128826.599
C_1-H_3	0.108	103040.767	0.110	44553.997
C_3-H_4	0.108	174987.164	0.109	160346.806
C_4-H_5	0.108	174972.285	0.109	160346.769
C_5-H_6	0.108	177194.675	0.109	162746.632
C_6-H_7	0.108	177195.752	0.109	162746.602
C_7-H_8	0.108	177695.924	0.109	163467.850
Bond angle	HF		MP2	
	θ_o (°)	k_θ (kJ/mol/rad^2)	θ_o (°)	k_θ (kJ/mol/rad^2)
C_1-C_2-C_3	120.891	235.101	120.816	166.314
C_1-C_2-C_4	120.860	235.062	120.816	166.489
C_2-C_3-C_5	120.990	238.825	120.817	179.752
C_2-C_4-C_6	120.991	238.518	120.977	179.754
C_3-C_2-C_4	118.240	408.253	118.977	211.767
C_3-C_5-C_7	120.219	261.361	120.338	207.856
C_4-C_6-C_7	120.219	261.068	120.107	207.856
C_5-C_7-C_6	119.341	365.274	119.107	203.478
H_1-C_1-H_2	107.611	262.262	108.495	136.390
H_1-C_1-H_3	107.976	204.014	107.082	163.676
H_2-C_1-H_3	107.619	229.373	107.695	180.226
H_1-C_1-C_2	111.278	146.042	111.028	186.814
H_2-C_1-C_2	110.908	136.360	111.028	182.659
H_3-C_1-C_2	111.280	144.634	111.160	108.718
C_2-C_3-H_4	119.565	84.937	119.445	71.724
C_2-C_4-H_5	119.561	84.928	119.445	71.724
C_3-C_5-H_6	119.724	99.254	119.811	76.539
C_4-C_6-H_7	119.727	99.248	119.811	76.539
C_5-C_3-H_4	119.445	98.751	119.578	76.323
C_6-C_4-H_5	119.449	98.762	119.578	76.323
C_5-C_7-H_8	120.330	99.226	120.252	77.688
C_6-C_7-H_8	120.328	99.229	120.252	77.702
C_7-C_6-H_7	120.054	87.592	120.082	75.873
C_7-C_5-H_6	120.057	87.611	120.082	75.873

Table 2: Bond stretch and bond angle force constants and equilibrium constants for a MOLD.

Bond stretch	HF		B3LYP		WB97XD	
	b_o (nm)	k_b (kJ/mol/nm^2)	b_o (nm)	k_b (kJ/mol/nm^2)	b_o (nm)	k_b (kJ/mol/nm^2)
C_1-C_2	0.154	59378.641	0.155	91021.542	0.154	97735.125
C_1-C_4	0.147	111358.175	0.153	94658.110	0.152	107782.771
C_1-N_1	0.140	134946.337	0.148	77825.379	0.147	117654.301
C_2-N_2	0.145	102898.923	0.146	115277.582	0.145	94802.287
N_2-C_3	0.144	123252.292	0.145	105553.018	0.145	105111.980
C_3-N_1	0.145	133053.768	0.146	93002.661	0.145	114547.475
N_2-C_5	0.145	135726.925	0.146	107684.979	0.145	116199.712
N_1-$C_1$1	0.145	141711.184	0.146	64328.327	0.145	130529.676
...						
$C_1$5-$C_1$6	0.156	61226.075	0.155	97598.267	0.153	95379.861
$C_1$6-O_3	0.125	169559.719	0.134	146722.655	0.134	123044.091
$C_1$6-O_4	0.121	191408.971	0.121	353759.366	0.121	103987.879
$C_1$5-$H_2$9	0.108	55304.396	0.110	106238.673	0.110	153883.152
$C_1$5-N_4	0.152	64744.492	0.148	92326.797	0.147	136728.429
N_4-$H_3$0	0.103	196128.777	0.186	-1001.003	0.187	2306.120
N_4-$H_3$1	0.101	105156.593	0.132	174195.469	0.101	173971.740
N_4-$H_3$2	0.101	95376.617	0.102	205622.255	0.102	211003.241
Bond angle	HF		B3LYP		WB97XD	
	θ_o ($^\circ$)	k_θ (kJ/mol/rad^2)	θ_o ($^\circ$)	k_θ (kJ/mol/rad^2)	θ_o ($^\circ$)	k_θ (kJ/mol/rad^2)
C_4-C_1-C_2	113.414	318.477	113.761	202.118	113.521	246.737
C_4-C_1-N_1	110.789	227.630	110.805	256.846	110.530	204.262
C_1-C_2-N_2	103.071	336.720	103.955	231.904	103.295	202.970
C_1-N_1-C_3	107.396	375.523	107.243	207.183	107.351	199.916
C_2-N_2-C_3	103.390	323.220	103.706	188.027	103.560	287.356
N_1-C_3-N_2	103.793	250.187	103.923	175.117	103.140	189.028
...						
O_4-$C_1$6-O_3	130.227	485.763	122.863	147.241	122.840	364.071
O_4-$C_1$6-$C_1$5	116.718	335.193	123.562	155.483	123.752	294.461
O_3-$C_1$6-$C_1$5	113.078	296.972	113.555	141.299	113.381	345.322
$C_1$6-$C_1$5-$H_2$9	110.803	327.222	106.992	191.918	106.851	121.548
$C_1$6-$C_1$5-N_4	104.186	238.229	107.843	265.474	107.914	181.744
$H_2$9-$C_1$5-N_4	108.172	279.257	112.879	238.263	113.399	95.011
$C_1$5-N_4-$H_3$0	102.658	111.257	83.991	21.584	83.786	51.992
$C_1$5-N_4-$H_3$1	114.182	238.440	111.317	234.889	111.440	135.813
$C_1$5-N_4-$H_3$2	112.234	251.319	111.236	91.949	111.517	213.310
$H_3$0-N_4-$H_3$1	111.791	102.048	133.172	40.772	130.976	43.035
$H_3$0-N_4-$H_3$2	107.028	121.242	107.185	31.9726	108.939	31.402
$H_3$2-N_4-$H_3$1	108.657	250.355	107.330	64.151	107.634	132.936

Table 3: Bond stretch and bond angle force constants and equilibrium constants for a zinc binding centre.

Bond stretch	HF		B3LYP		B3PW91	
	b_o (nm)	k_b (kJ/mol/nm^2)	b_o (nm)	k_b (kJ/mol/nm^2)	b_o (nm)	k_b (kJ/mol/nm^2)
N_1-Zn	0.208	24972.189	0.207	38186.498	0.206	29314.894
N_2-Zn	0.207	10120.548	0.207	12537.478	0.206	15342.023
N_3-Zn	0.205	29186.807	0.208	27360.235	0.207	21777.239
O_1-Zn	0.195	32679.279	0.201	25746.753	0.200	26324.168
Bond angle	HF		B3LYP		B3PW91	
	θ_o (°)	k_θ (kJ/mol/rad^2)	θ_o (°)	k_θ (kJ/mol/rad^2)	θ_o (°)	k_θ (kJ/mol/rad^2)
N_1-Zn-N_2	110.014	182.932	111.471	134.866	111.525	249.867
N_3-Zn-O_1	97.290	159.586	94.660	94.259	94.314	89.958
N_4-Zn-N_3	115.731	87.810	114.569	103.374	114.508	124.739
N_2-Zn-O_1	106.721	91.759	114.600	96.787	115.321	94.642
N_2-Zn-N_3	109.443	122.018	108.803	98.390	108.712	103.314
O_1-Zn-N_3	116.828	167.950	112.330	67.774	112.056	66.996

Table 4: Laplacian bond order for toluene.

Bond	HF	MP2
C_1-C_2	1.284794	1.306510
C_2-C_3	1.795650	1.748262
C_2-C_4	1.794852	1.748267
C_3-C_5	1.825264	1.781232
C_4-C_6	1.826044	1.781231
C_5-C_7	1.831004	1.783064
C_6-C_7	1.830184	1.783067
C_1-H_1	0.863936	0.839790
C_1-H_2	0.846973	0.839797
C_1-H_3	0.863425	0.824890
C_3-H_4	0.886123	0.853989
C_4-H_5	0.886034	0.853990
C_5-H_6	0.892607	0.861999
C_6-H_7	0.892625	0.861998
C_7-H_8	0.888990	0.858737

Table 5: Laplacian bond order for MOLD.

Bond	HF	B3LYP	WB97XD
C_1-C_2	1.221273	0.987163	1.023464
C_1-C_4	1.265259	1.047901	1.071812
C_1-N_1	0.866105	0.660360	0.694804
C_2-N_2	0.954822	0.732890	0.772124
N_2-C_3	1.030577	0.799955	0.831084
C_3-N_1	0.994014	0.781099	0.803401
N_2-C_5	0.947139	0.743520	0.772003
N_1-C_{11}	0.963764	0.750147	0.785709
...			
C_{15}-C_{16}	1.215368	1.026716	1.077801
C_{16}-O_3	1.234298	0.618620	0.632717
C_{16}-O_4	1.445232	1.250907	1.299047
C_{15}-H_{29}	0.896539	0.782734	0.791491
C_{15}-N_4	0.680474	0.724798	0.760664
N_4-H_{30}	0.686890	NA	NA
N_4-H_{31}	0.792246	0.705839	0.708033
N_4-H_{32}	0.793018	0.711315	0.720225

Table 6: Laplacian bond order for a zinc binding centre.

Bond	HF	B3LYP	B3PW91
N_1-Zn	0.260113	0.243007	0.251249
N_2-Zn	0.267039	0.241378	0.247556
N_3-Zn	0.264694	0.233681	0.240049
O_1-Zn	0.166463	0.137009	0.138954
